# Assessing the Efficacy of Contextualized Group Counseling Education in Asia: A Mixed Methods Study

**DOI:** 10.1007/s10447-022-09471-3

**Published:** 2022-06-09

**Authors:** Rachel Sing-Kiat Ting, Justine Jian-Ai Thong, Joy Yung-Re Lim, Elizabeth Jones

**Affiliations:** 1 Department of Psychology, Monash University, Bandar Sunway, Selangor Malaysia; 2 Monash University, 47500 Bandar Sunway, Selangor Malaysia

**Keywords:** Group counseling, Experiential learning, Training efficacy, Contextualization, Asian culture

## Abstract

Many experiential learning teaching models are developed in Western cultures, with their efficacy not tested in non-Western cultures, especially in counseling education. This study examined the learning experiences of students (n = 52) enrolled in a culturally contextualized experiential teaching method implemented across a 3-year period in a Malaysian university Masters-level group counseling course. The course changes included group demonstrations by lecturers and peers, live group participation and observation, group dynamics map drawing and debriefing, paired group proposal writing and presentation, and group note- taking. This study adopted a mixed method approach with a short survey consisting of both standardized scales and open-ended questions, administered at the beginning and end of each 12-week semester. Archival data on students’ evaluation was also retrieved from the year before the intervention, to compare with the 3 years of intervention. Results showed a statistically significant improvement in students’ perceived group leadership skills and leadership characteristics at the end of the course. Students’ overall satisfaction with the course quality also improved significantly from the year prior to the implementation of the new teaching method. Qualitative coding identified three major themes–active learning classroom that led to confidence in group counseling knowledge and skills; experiential activities in the tutorial sessions, with opportunities to carry out their proposed group activities; and formative feedback given throughout the semester during tutorial settings contributing to the higher course satisfaction rate. To conclude, we discuss the implications of contextualized experiential learning for higher education counseling pedagogy in the Asian region.

**Supplementary information:**

The online version contains supplementary material available at 10.1007/s10447-022-09471-3.

## Assessing the Efficacy of Contextualized Group Counseling Training in Asia

As globalization leads to the development of new mental health needs and counseling theories, the training of counseling professionals requires ongoing attention and improvement (Leong & Leach, 2008). Group counseling training, in particular, has been better established in the Western and developed countries, while training programs in Asia still contend with a lack of empirically-validated models and approaches. This limitation to group counseling pedagogy in Asia is a significant one, as learning styles vary across cultures (Liu, 1998; Watkins & Regmi, 1996), suggesting that group counseling training models may need to be adapted to an Asian context.

To date, the inclusion of experiential elements (Kolb & Fry, 1975; Kolb, 1984**)** is considered a core component in most counselor training programs, whereby trainees are typically required to participate as group members in an approved small group activity for a minimum of ten hours over one academic term (Council for Accreditation of Counseling and Related Programs [CACREP], 2016, p. 11). Likewise, during practicum, trainees are required to engage in leading or co-leading a group session (CACREP, 2016, Sect. 3: E). Research has demonstrated that experiential learning elements in counseling training programs improve counseling skills among trainee practitioners (Chang et al., 2016; Paladino et al., 2011). In addition to improving technical skills, group experiential activities have been found to facilitate self-awareness, personal growth, meaningful interpersonal experiences, multicultural and professional identity, and cognitive complexity among trainee counselors in American training programs (Anderson et al., 2014; DeDiego, 2016; Smith & Davis-Gage, 2012; Little et al., 2005).

However, not all studies have found positive effects for experiential learning activities across the board. For example, in a study of psychoeducation versus personal growth-oriented experiential group training models, neither group demonstrated improvements in cognitive and affective empathy (Ohrt et al., 2013). Another study with counselling trainees from a Lithuanian university found that trainees experienced confusion and lack of clarity when undertaking group experiential training, which impeded their development of group leadership skills (Jakubkaitė & Kočiūnas, 2014). Moreover, there has been limited research on the effectiveness of experiential learning in non-Western cultures, which is the focus of the current study. In the past, effectiveness of group counseling training was measured through other-report (e.g. supervisor; see Ahmad et al., 2018) or self-report from the trainees on their level of self-efficacy, self-awareness and skills (see Flasch et al., 2011). In this study, we focused on the latter training outcome, specifically the self-confidence in “leadership characteristics” and “leadership skills”, as these are the essential components that are associated with successful group leaderships (Corey et al., 2017). In addition, we also included the level of trainees’ satisfaction towards the training curriculum, as this is a core indicatormany universities use to assess pedagogical success.

## Counselor Training in Malaysia Context

Counselor training in Malaysia was introduced in the 1960s, with influences from the American counseling system (Amir & Latiff, 1984). From the 1980s, more comprehensive training requirements for counselors were implemented (Othman & Aboo Bakar, 1993) following the formation of the Malaysian Counseling Association, known as PERKAMA (Persatuan Kaunseling Malaysia) and the enactment of the Counsellors Act 1998 (Act 580; Commissioner of Law Revision and Percetakan Nasional Malaysia Bhd, 2006) in the 1990s. The curriculum of counseling training for both Bachelor and Master degrees requires that all students partake in a group counseling course as part of the Core Competencies units for clinical training. Succesfully training counselors to lead counseling groups is important, as group counseling was demonstrated to be effective in a strong-ties society, such as Malaysia and especially in medical (Alvani et al., 2015; Ting et al., 2018) and mental health settings (Mukhtar et al., 2011; Phang et al., 2016; Wan Sulaiman et al., 2017). For example, a phenomenological study by Bakar et al., (2020) found that mental health patients in a local Malaysian psychiatric clinic benefited from social support and social skills learning through group counseling sessions. Similarly, a study of 57 substance abuse clients in a residential drug treatment clinic in Malaysia by Sabri et al., (2017) found that participants’ treatment outcomes and psychological well-being improved significantly following solution-focused group therapy. Thus, identifying effective approaches to developing the skills of counselors to lead group sessions in the Malaysian healthcare context is important.

However, there has been almost no research into the effectiveness of group counseling training methods in Malaysia. Ahmad and his colleagues (2018) carried out an action-research study with 35 postgraduate students in a public university of Malaysia, and found that integration of traditional teaching methods and experiential learning (such as mind mapping and role-play) resulted in effective and practical learning outcomes. However, their findings were primarily based on qualitative data collected through informal peer and lecturer feedback, and students’ diaries, without rigorous coding procedures. More evidence is required regarding the link between experiential training modules and student learning outcome.

In our study, we drew on the ecological rationality theory (Todd & Gigerenzer, 2012), which espouses that societal mentalities co-evolve together with their environment, and are useful for the ecological niches wherein they originally evolved to function in. Taking this perspective into the theoretical construction of indigenous psychology, Sundararajan (2020) explained rationalities (cognitive styles) differ across cultures, with strong ties versus weak ties as ecological niches (Granovetter, 1973). To illustrate, before globalization, human societies evolutionarily developed as close-knit groups where narrow but strong relationships (strong ties) and relational cognition (Oishi & Kesebir, 2012) were functionally optimal for wellbeing and happiness (Buss, 2000). While Malaysia has undergone modernization over the past half-century since its independence from being a British colony (Sumaco et al., 2014), relatively strong-tie society characteristics still remain, such as relational-focused cognition, social interdependence, and social hierarchy (Abdullah & Pederson, 2003; The Hofstede Centre, n.d.). In contrast, weak-tie societies lean toward mind-to-world transactions, transactions best served by non-relational cognition (Sundararajan, 2015). As such, the pedagogical task of highlighting relational cognition in group therapy training programs in Asian regions such as Malaysia is needed. To fulfill this necessity, we developed a contextualized model of group counseling education by incorporating experiential exercises (the relational component) using group demonstrations, fishbowl observations, and debriefing sessions to scaffold the meta-knowledge gained from their relational experiences.

## Pedagogy Design

This study took place in an international university in Malaysia. The student body in this campus was culturally diverse and included students from a variety of countries, predominantly from Southeast Asia. The university’s Master of Professional Counseling program was established in 2016 with accreditation from both a local professional body and the Australian professional association. The Group Counseling unit was a 12-week long course, which first year students completed as a part of their core clinical competencies prior to the commencement of their practicum. The learning objectives of the course were as follows: (a) understand the suitability and strengths of group therapy; (b) apply diverse theories of group dynamics and interpersonal roles in leading group counseling processes; (c) demonstrate the documentation skills for professional group counseling; (d) identify the challenges and ethical issues associated with group counseling; (e) develop sensitivity to the multicultural nature of group counseling in Malaysia; and (f) reflect on how to be an effective group counselor.

Previously, the curriculum was mainly lecture-based, with 10 weeks of didactic teaching and 2 weeks of unstructured practice of group demonstrations, held towards the end of the semester. The assessment tasks were theoretical essays to critically review literature on group counselling models and a self-appraisal of therapeutic practice as an individual client in a group. Based on an anonymous teaching evaluation in 2017, the majority of students reported overall dissatisfaction with this course, in particular the lack of clarity regarding assessment tasks, a lack of active class participation, unclear linkage between learning activities and learning outcomes, insufficient feedback, and minimal opportunities for critical thinking. Therefore, in 2018, the course was redesigned through the adoption of the active-experiential learning approach (Kolb, 1984; Kolb et al., 2001; Jenkins & Clarke, 2017; Yalom & Leszcz, 2005), with the integration of lecture and tutorial activities.

**Fig. 1:**
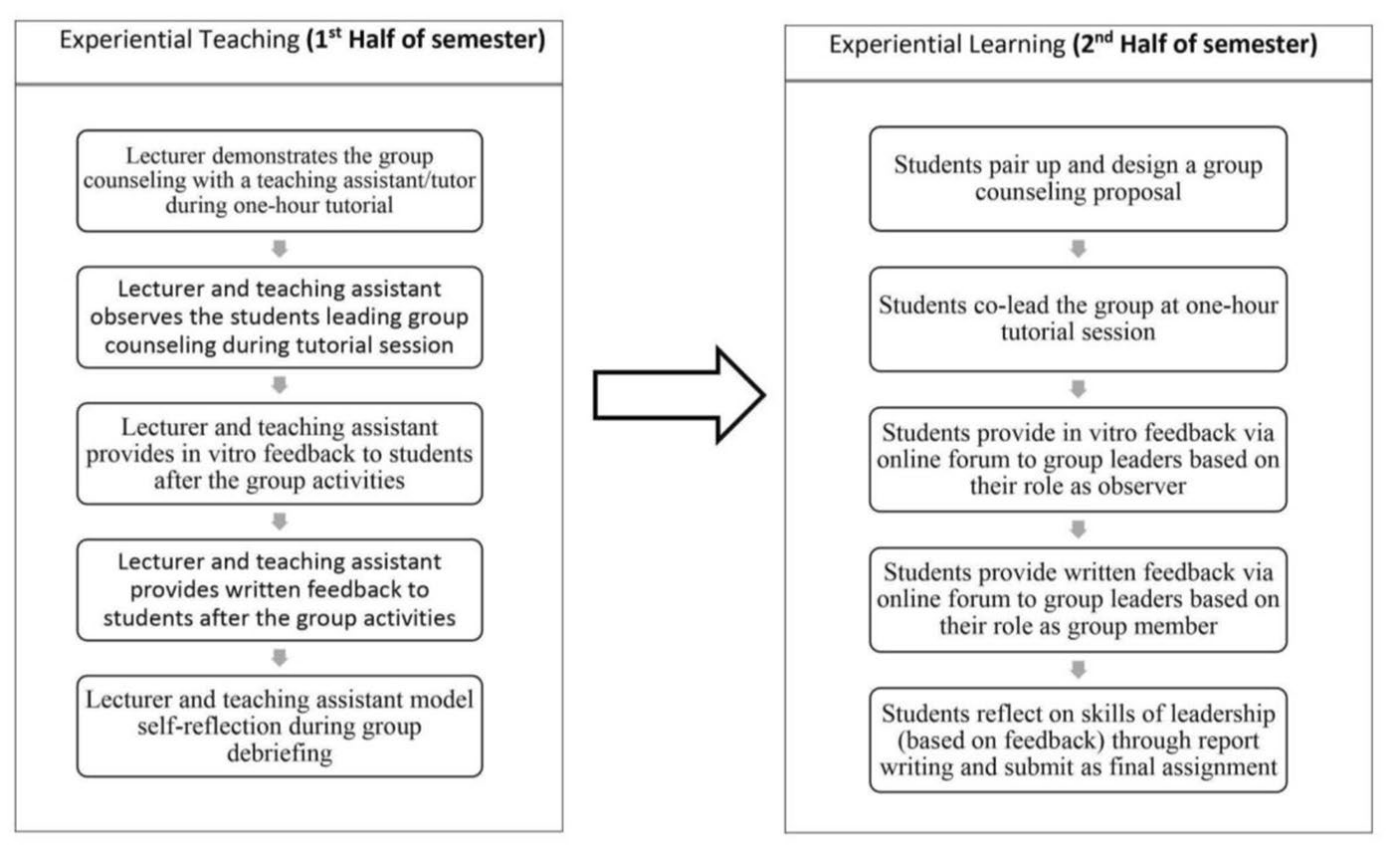
Flow Chart of Summarized Activities for One Semester

Over the 12 weeks, there were one-hour weekly lectures on theories and models of groups, group dynamics, group roles, multiculturalism, and group development. These lectures were supplemented with two-hour small group (10–12 students) tutorial activities. Throughout the 12-week semester, the experiential learning approach, with practice-reflection loops, was delivered via four key components of classroom activities: Group demonstration sessions, Debriefing sessions, Observation sessions, and Group documentation writings (See Fig. 1).

1. Group Demonstration sessions. In weeks 1–2, students were exposed to “Color of Fear” (a reality multicultural group documentary by Lee, 1994), and engaged in reflection and discussion with the lecturer regarding issues of multiculturalism in their respective home-country. In weeks 3 and 4, a 30-minute “fishbowl” demonstration (Kane, 1995) was conducted by the lecturer or tutor, for the purpose of modelling group counseling leadership. In weeks 6–8 students were invited to voluntarily role-play as group members, while the rest were “fishbowl” observers. The lecturers demonstrated group counseling skills (such as linking, questioning, empathizing, guiding, here-and-now intervention, etc.) through structured activities. Typical activities included a “power walk” in the first session to raise awareness regarding “power and privilege” and enable students to build rapport with each other through personal disclosure. To avoid the dual-relationship dilemma in this type of experiential activity (Shumaker et al., 2011), students were told to “create a new identity” and to experiment by utilizing different social roles spontaneously, which might be either dysfunctional or functional to the group’s process.
2. Debriefing sessions: The group demonstration sessions were usually followed by 30-minute debriefing sessions, where the lecturer processed the “fishbowl” with student observers and participants, relating their reactions to social roles and group theories, and mapping of the group dynamics visually.
3. Group observation sessions: In the second half of the semester, students were paired as co-leaders to design and conduct a mock group counseling session with their peers during tutorial sessions (10–12 students). They were also observed by their peers and lecturers during their group’s demonstration. The lecturer then debriefed group participants, observers, and leaders on their respective roles. Students reflected on their leadership experiences in a structured and intentional way, while their peers provided constructive feedback on their leadership skills.
4. Students’ observation and reflection reports: Assignments, including two group observation reports and two reflective leadership reports, were an integral part of the assessment in this course, for the purpose of conceptualizing their experiential learning using theoretical frameworks. The lecturers also provided written feedback on these assignments to facilitate further growth in the students’ knowledge and skills of group counseling.

In the year 2020, due to the Covid-19 pandemic situation and the ensuing nationwide lockdown measures, the course was converted to a blended mode of delivery where 30% of the group demonstrations were undertaken online via the Zoom digital platform.

## Research questions and hypotheses

Data was collected first to answer the research question of whether the revised teaching model would meet the learning outcomes for the course of developing group leadership skills and characteristics. Second, we aimed to examine whether students were satisfied with the new curriculum for this course, and what elements of the course were helpful for their learning. We posed the following hypotheses:

### Hypothesis 1

There would be a statistically significant increase in Group Leadership Characteristics and Group Leadership Skills from the start to the completion of the course.

### Hypothesis 2

There would be a statistically significant increase in students’ course satisfaction (SETU score) in 2018–2020 (post-implementation) when compared to 2017 (pre-implementation).

In addition, we elicited open-end feedback from students regarding the course elements that they found most helpful.

## Method

### Study Design and Procedure

This was a mixed-methods survey study using both standardized scales and open-ended text fields. Hypothesis 1 was tested using data obtained through surveys implemented from 2018 to 2020 and hypothesis 2 through the archival data of the university’s course evaluations from 2017 to 2020. The survey was administered to student participants at two different time points of the semester (first two weeks of class and after the final week of class) from 2018 to 2020. Ethics approval was obtained from the University Human Research Ethics Committee. All students from the Master of Professional Counselling program (2018–2020) who were enrolled in the Group Counselling course were approached by an independent research assistant during the first 2 weeks of semester. In 2018 and 2019 the paper-based survey was administered physically by a research assistant who held a Bachelor in Psychological Science and had no conflict of interest with the potential participants. In 2020, due to the pandemic lockdown, the survey was administered to interested participants online via an email. Participation was voluntary and the survey deidentified. The lecturer did not attend the briefing session, run by the research assistants, where students were recruited into the study, and was also blind to the participation rate until the course and all assessment was completed. To protect participants’ confidentiality, they were asked to use a pseudonym on their surveys. A token of appreciation was offered to 10 participants through a lottery draw for a gift voucher worth 50 Ringgit Malaysia (equivalent to 12 USD).

All classes were conducted by the 1st author, a clinical psychologist with extensive teaching and clinical experience in group facilitation.

### Participants

The survey data were collected from Masters-level students who were enrolled in the Group Counseling course during 2018–2020. Across the 3 cohorts an estimated 120 potential participants were approached by the research assistants. Eighty students (66% of eligible students) consented to participate and proceeded to complete the Time 1 pre-course survey, with 65% of participants from Time 1 completing the Time 2 post-course survey. The mean age of participants was 26.08 (SD = 6.34). A total of 52 participants, consisting of 21% male (n = 11) and 79% females (n = 41), completed both parts of the survey. Other key demographic information, including participants’ ethnicity and nationality, are presented in Table 1.

**Table:** Table 1 Summary of Participants’ Demographic Background (n = 52)

Variables	*n*	(%)
**Gender** Male Female	11 41	(21.2) (78.8)
**Ethnicity** Chinese Indian Bangladeshi Bidayuh Caucasian African	39 7 1 1 1 1	(76.9) (13.4) (1.9) (1.9) (1.9) (1.9)
Eurasian	1	(1.9)
Non-stated	1	(1.9)
**Nationality** Malaysians Bangladeshi Indian British Mauritian Republic of Chinese Sri-Lankan Tanzanian Egyptian	42 2 2 1 1 1 1 1 1	(80.8) (3.8) (3.8) (1.9) (1.9) (1.9) (1.9) (1.9) (1.9)

The archival data were collected anonymously by the university without collecting any background information on the students, thus we cannot provide any demographic information regarding these participants. Upon the request of the authors a total of 111 individual SETU scores for the group counseling course was released across years 2017 (n = 16), 2018 (n = 32), 2019 (n = 31) and 2020 (n = 32) by the university’s Unit Evaluations office. This sample thus may or may not include the same participants as used to test hypothesis 1.

### Measures

The survey consisted of three parts: Demographic questions, competence in leading group counseling, measured by the Self-Assessment of Group Leadership Skills (SAGLS) scale; and personal leadership characteristics, measured by the Group Leader Characteristics Self-Report Scale (GLCSRS).

#### Self-Assessment of Group Leadership Skills (SAGLS)^1^

There is currently no validated measure of group leadership skills for counseling. Thus, the self-assessment of group leadership skills questions was developed based on Corey et al.’s (2017) “Theory and Practice of Counseling and Psychotherapy” textbook, which was the main prescribed reading for this course, and addressed the learning objectives for the course. Participants were asked to rate their competence on 18 items measuring different aspects of group leadership micro-skills, such as “Reflecting: Capturing the underlying meaning of what is said or felt and expressing this without being mechanical”, “Summarizing: Identifying key elements and common themes and providing a picture of the directional trends of a group session”, and “Empathizing. Adopting the internal frame of reference of a member”. The items were rated on a 3-point Likert scale, ranging from *I do this occasionally with a relatively low level of competence* (1) to *I do this most of the time with a high degree of competence* (3). Participants’ ratings were averaged to provide an overall score ranging from 1 to 3, with higher scores indicating higher self-reported competence. The scale had good reliability at both time points with a Cronbach alpha of 0.86 for the pre-course survey and 0.79 for the post-course survey in this study.

#### Group Leader characteristics Self-Report Scale (GLCSRS)^2^

Although there are many theories of leadership, most of the scales developed assess leadership styles, rather than the personal characteristics or qualities of a group leader in a counseling setting. Some personal characteristics have been widely agreed to build competent leadership, such as intelligence, openness to new experience, and problem-solving skills (Fassinger & Shullman, 2017). Therefore, we developed a measure based on the model of group leadership characteristics proposed by Corey et al., (2017). The measure consisted of 12 questions assessing effective group leadership characteristics, such as “I have good intentions, sincerity, respect, and trust towards other people”, “I can share my feelings with others and connect with group members emotionally”, “I am willing to be a role model for the group members, to set good examples for them”, and “I have a good sense of humor”. Participants rated the items on an 11-point Likert scale, ranging from *totally not me* (0) to *totally is me* (10). Each participant’s ratings were averaged to provide an overall score ranging from 0 to 10. In this study, internal consistency was high for both the pre and post surveys (Cronbach alpha = 0.90).

#### Open-ended questions

To collect qualitative data, in the post-course survey participants were also asked four open-ended questions about their perceptions of the course: “What have you found helpful in this class?”, “What do you think could be improved in this class?”, “How was your experience in the tutorial session?”, and “What can be improved in the tutorial session?”.

#### Student evaluation of teaching and units (SETU)

To test hypothesis 2, we utilized archival data collected annually by the university, which is administered at the end of each semester to measure students’ satisfaction with their enrolled courses. Ten items were included in this SETU anonymous online survey, measuring student’s self-reported active learning, critical thinking, perceived achievement of learning outcomes, perceived efficacy of assessment methods, perceived usefulness of the feedback and classroom activities, and satisfaction with the course. All items were rated on a 5-point Likert scale, *strongly disagree* (1) to *strongly agree* (5).

## Results

To examine the relationship to other variables for the scales, the authors tested the relatinoship between SAGLS and GLCSRS using a Spearman correlation analysis. The spearman rho correlation analysis indicated that there was a statistically significant positive association between SAGLS and GLCSRS in both the pre- and post-intervention (r = .39 ~ .57, *p* < .01). The moderate level of correlation indicated that these two constructs are related but independent (trait versus skill), and their convergence became stronger after the effective training. Prior to conducting inferential analyses, data was cleaned and checked. There were no multivariate outliers, and assumption of multivariate normality was met using the Shapiro-Wilk test as a reference. Finally, there was no multicollinearity, as all VIF values ranged from 1.59 to 8.38.

### Hypothesis 1

**Increased Group Leadership Characteristics and Group Leadership Skills after Course completion**

Paired sample *t*-tests analyses were conducted to compare participants’ average ratings of their group leadership characteristics and group leadership skills in conducting group counseling before and after completing the course. Results showed that (a) participants’ post-GLCSRS scores (M = 6.9, SD = 1.2) were statistically significantly higher than their pre-GLCSRS scores (M = 6.4, SD = 1.3), t (51) = 2.49, *p* < .05; *d* = 0.34 and (b) their post-SAGLS scores (M = 2.1, SD = 0.3) were statistically significantly higher than their pre-SAGLS score (M = 1.8, SD = 0.3), t (51) = 6.31, *p* < .001; *d* = 0.89. Hypothesis 1 was confirmed with medium to large effect sizes (Cohen, 1988).

### Hypothesis 2

**Increased course satisfaction after implementation of experiential learning**

As Table 2 showed, an independent one-way Multivariate Analysis of Variance (MANOVA) was conducted to compare 10 SETU items, across four student cohorts (2017 = control group; 2018, 2019, and 2020 = cohorts with implementation of experiential learning). As sample sizes differed across the four years [2017 (n = 16), 2018 (n = 32), 2019 (n = 31), and 2020 (n = 32)], Pillai’s coefficient was reported for the MANOVA, due to its higher stringency for homogeneity of covariance matrices (Tabachnick & Fidell, 2013). MANOVA results showed a statistically significant difference in SETU scores across the years, *F* (30, 288) = 2.98, *p* < .001; Pillai’s *V* = 0.710. The partial eta-squared (η^2^ = 0.237) indicated a large effect size (Cohen, 1988). Post hoc comparisons, using the Tukey HSD test, confirmed that the mean scores for all ten items in 2018, 2019, and 2020 cohorts were statistically significantly higher (*p* < .001) than the 2017 cohort (See Table 3). The key improvement occurred between 2017 and 2018, the year after implementation of experiential learning. In addition, the significant improvement carried on in 2019, the second year of implementation and plateaued in 2020. Hypothesis 2 was thus confirmed.

**Table 2:** Results comparing SETU items scores for years 2017 (pre-experiential learning) and 2018–2020 (experiential learning)

Items	2017 (n = 13)		2018 (n = 32)		2019 (n = 30)		2020 (n = 32)		
	M	SD	M	SD	M	SD	M	SD	F (3, 103)
1. The Learning Outcomes for this unit were clear to me	2.85^a^	1.34	3.94^b^	0.72	4.40^c^	0.50	4.47^c^	0.72	16.01***
2. The instructions for Assessment tasks were clear to me	2.23^a^	1.24	3.56^b^	1.13	3.60^b^	1.15	3.66^b^	1.26	5.10***
3. The Assessment in this unit allowed me to demonstrate the learning outcomes	2.31^a^	1.38	3.84^b^	1.14	4.47^c^	0.68	4.34^c^	0.60	18.91***
4. The Feedback helped me achieve the Learning Outcomes for the unit	2.15^a^	1.21	3.63^b^	1.34	4.43^c^	0.50	4.19^c^	0.86	17.55***
5. The Resources helped me achieve the Learning Outcomes for the unit	2.62^a^	1.45	3.53^b^	0.76	4.27^c^	0.69	4.09^c^	0.93	12.25***
6. The Activities helped me achieve the Learning Outcomes for the unit	2.54^a^	1.39	3.88^b^	0.94	4.57^c^	0.57	4.44^c^	0.91	17.34***
7. I attempted to engage in this unit to the best of my ability	3.46^a^	1.27	3.94^a^	0.80	4.37^b^	0.67	4.41^b^	0.61	5.98***
8. Overall, I was satisfied with this unit	2.23^a^	1.36	3.31^b^	1.38	4.23^c^	0.77	4.19^c^	0.90	13.63***
9. This unit helped improve my capacity for critical thinking	2.31^a^	1.32	3.81^b^	0.78	4.27^c^	0.58	4.22^bc^	1.04	16.73***
10. I was encouraged to actively participate in this unit	2.46^a^	1.45	3.69^b^	0.86	4.40^c^	0.62	4.47^c^	0.67	21.39***

*Note*. ***p **≤** .001; a, b, c: different letters mean significant difference at post-hoc paired comparison, whereas the same letter means no significant difference between the paired comparison

**Table 3:** Themes and Subthemes for Helpful Aspects of the Course

Themes	Sub-themes	Frequency (%)
Active learning classroom		34 (65.4)
	Gaining group leadership skills and group dynamic knowledge	9 (17.3)
	Classroom structure	8 (15.4)
	Group counseling demonstration videos	8 (15.4)
	Lecturer’s teaching experiences	7 (13.5)
	Interaction in classroom	6 (11.5)
	Practical coursework and assignments	6 (11.5)
	Comfortable classroom and dynamic	5 (9.6)
Experiential tutorial activities		30 (57.7)
	Group demo/role-play as participants and observers	19 (36.5)
	Group leadership experiences	9 (17.3)
	Interactive and engaging tutorials	8 (15.4)
Feedback loop		8 (15.4)
	Feedback from the lecturer/tutor	8 (15.4)
	Feedback from peers	4 (7.7)

## Qualitative Analysis

Qualitative feedback was provided by 52 students via open-ended questions in the post-course survey. Inductive thematic analysis (Braun & Clarke, 2006) was used to code responses to the question “what do you find helpful in this course?”. Two authors with training in qualitative coding (JTJA and JYRL) followed a consensual qualitative approach (CQR) (Hill et al., 1997), with further evaluation by an auditor (RTSK, the lecturer for the course) for internal consistency. More specifically, following the generation of initial codes independently by the two coders, themes were derived collaboratively by the same two coders. Any disagreements between the coders were resolved through discussion with the auditor. As shown in Table 3, three major themes were identified–*Active learning classroom*, *Experiential tutorial activities*, and *Feedback loop*.

*Active learning classroom.* This feedback targeted the curriculum design, learning outcomes, lecture delivery modality, assessment scheme, and the lecturer’s teaching skills. A majority of students (65.4%) reported that these *Academic related activities* were beneficial for their learning experience, which included *learning of group leadership skills, interactive lectures, the lecturer’s experiences*, as well as *relevant coursework and assignments*. For instance, one student commented that “the lectures addressed skills required for group counseling, the factors to consider when observing or leading a session, the roles members and leaders play, and the ethical issues and emergency situations to watch out for. This was very comprehensive and prepared us for roles as group leaders in the future”. Despite being converted to blended teaching in 2020, one student commented it was a “very interactive and interesting class, though it was conducted online”.

*Experiential tutorial activities.* This theme described feedback targeting the experiential group activities during tutorial sessions that were participatory in nature and not directly assessed. More than half (57.7%) of the students identified the usefulness of the experiential activities, including the various group demonstration activities, and their roles as group leaders, members, and observers. For example, one of the students said “being a group participant led by different group leaders let me see different styles and different personalities”, while another referred to how “experiential learning really helped to understand the content and application of the teaching”. The integration of the report-writing skill practice was also found helpful,with one student stating that “the live demonstration of our group session was also a good experience along with report-writing which I think is important in conducting group sessions”.

*Feedback loop.* Some students (15.4%) also indicated that a feedback loop, consisting of instant feedback and discussion with lecturers, tutors and peers after the group demonstration session, was helpful. One student wrote, “The lecturer’s feedback, ‘here and now’ examples were relevant and can be easily understood”.

When asked about suggestions for improvement, many students suggested having a smaller lecture class size, more coaching in report writing, and more opportunities to practice their group leadership skills. For instance, students requested more experiential role-play opportunities, “more role playing and deeper roles…”.

## Discussion

Our study indicated that a contextualized experiential learning approach was both acceptable to trainees and efficacious in developing their self-assessed leadership skills and qualities in a non-Western context. After a semester of participation in the course, counseling students demonstrated improvements in both their leadership skills and characteristics in a group counseling context; moreover their satisfaction with the course increased. From a developmental perspective, the increased self-confidence in trainees may benefit the development of their mental health professional identity as well (Stoltenberg & McNeill, 2011).

Previous research indicated mixed results for the effectiveness of experiential learning (Bore et al., 2010; Jakubkaitė & Kočiūnas, 2014), particularly in Eastern cultures. Our contextualized approach demonstrated that experiential activities can be effective for beginner trainees, particularly where there is also a clear structure in the curriculum. According to the Ecological Rationality Framework, different cognitive processing channels may be privileged by different cultures (Sundararajan, 2020), with strong-ties societies, such as Asian cultures, preferring more relational and concrete cognition. Experiential learning could thus be a good fit for Asian mentalities because of their sensitivity in relationship, but we suggest that this should be balanced with the concrete structure of an active classroom, such as through brief lectures and the use of a feedback loop from the lecturer on students’ written assignments. Pure experiential exercise may be too risky and confusing for Asian students, as it is not a social norm to be emotionally expressive in the classroom. In the past, Asian students’ learning styles are thought to be influenced by Confucianism, where the application of examples and rote learning are long standing in learning and teaching (Chan, 1999). Yet, our study found that students were open to experiential learning when given the opportunity, and requested more of it in their feedback, as seen in our qualitative findings.

In a globalized era, when addressing the need of a culturally diverse student population, we argue a blended approach, which integrates both experiential activities and structured academic related activities, is needed in the training of counseling students. As per the movement of “internationalizing counseling psychology” in the Asian region (Leong & Ponterotto, 2003), an experiential approach appeals to the cognitive styles of many Asians who embrace holistic thinking and learning (Nisbett & Miyamoto, 2005). At the same time, structured learning activities, such as report-writing and engaging lectures would also be essential for students in acquiring knowledge and theories of group counseling. Our qualitative findings demonstrated that student participants valued both the academic and experiential activities.

However, this method of teaching is labor intensive. More than one teaching assistant was required to provide timely feedback on assignments and experiential activities for a class size of 40–50. Group facilitators also require intensive training to be able to guide students through the experiential debriefing and to provide quality feedback. Universities need to consider investing in training qualified teaching assistants, or employing adjunct faculty (such as professional counselors or clinicians), to assist in conducting experiential activities for optimal outcomes. As online or blended learning is increasingly the trend in college education (Bashir et al., 2021), including counselor training programs, video recordings of group demonstrations may be helpful for students’ self-learning. Furthermore, as online group counseling becomes the new normal (Situmorang, 2020), incorporating topics on digital mental health ethics and emphasizing multicultural group techniques in counseling training would be apt.

## Limitations and Future Research

There were a number of limitations to our study. First, while participation in the pre-course survey was high, there was a fairly high rate (35%) of non-completion of the post-course survey. The attrition rate could be caused by “evaluation fatigue” at the end of the semester, where normally students were asked to provide other forms of university evaluation on teaching. End of semester is also usually a stressful time for the students due to assignment deadlines. In addition, for the first 2 years we administered the survey in a paper-based format, hence finding a suitable time for the students to meet the research assistants was challenging. Administering the survey in a digital format would allow for greater flexibility in timing to complete the survey, which may improve the response rate. In 2020, the unexpected pandemic situation may also have affected the response rate from the students.

Second, due to the lack of validated group leadership skill-based measurements, both scales used in this study were developed for the current study based on the skills and characteristics identified in a key group counseling textbook by Corey et al., (2017). We note that previous published studies (e.g., Erikson et al., 2016; Medina et al., 2019; Nazir et al., 2020) also primarily relied on lecturers’ reports, students’ reflective journals or teaching evaluation as measurements of the efficacy of the course. As the operationalization of group leadership adopted in this study was from a Western pedagogy (e.g. Corey et al., 2017), the scales adopted may not be the most valid measure of group leadership skills in a non-Western society. Hence, there is a need for future studies to develop culturally-validated measures of leadership skills for the group counseling context.

Third, we used self-report ratings of students’ leadership skills and qualities. Though the SAGL items were rated based on behavioural frequencies, the Likert scale created did not allow for a “0” option, to indicate “never practice at all”, and had a limited range. We expected students to have some skills prior to commencing the course, based on the selection criteria for the program, however future studies could increase the rating to 5 points or 7 points, to allow for more variability, as well as potentially including lecturer ratings as part of the outcome measures.

Fourth, there are some factors to consider regarding the delivery of the course. The group counseling course was carried out in a “blended” mode of learning in 2020. The pattern of results was similar across years, suggesting the efficacy of online delivery for Malaysian students. Future research could test this assumption by comparing different modalities of delivery (online, blended versus face-to-face). There was also a change in the lecturer during the intervention (2018–2020), compared to 2017, which may have affected the results, although both lecturers were experienced educators and counsellors.

Lastly, as we did not have a comparison group or control group for this study, it is hard to differentiate between the effectiveness of a purely experiential learning versus a contextualized experiential learning. Future researchers may consider comparing multiple experiential learning approaches to further examine the role of cultural differences in preferred learning styles.

## Conclusions

Our study examined the effectiveness of experiential teaching that was adapted to the Malaysian counselor education context. Both quantitative and qualitative data showed that students benefited from a contextualized approach that catered to their cognitive style through concreteness in the classroom structure and observation and participatory experiences in the tutorial sessions. Three years of data consistently demonstrated students’ growth in professional identity and leadership skills, including when converted to an online blended mode in response to the pandemic. Cultural sensitivity arguably plays a vital role for educators in non Western regions when translating experiential learning into the local context. Our study is not without limitations, yet it reveals promising results for the cultural integration of active learning into Asian classrooms.

## Electronic supplementary material

Below is the link to the electronic supplementary material.

Supplementary Material 1

## Data Availability

The data that support the findings of this study are available on request from the corresponding author. The data are not publicly available due to their containing information that could compromise the privacy of research participants.

## Footnotes

1 the scale is available by contacting the authors

2 the scale is available by contacting the authors
