# Supplementary material for: Assessing the Efficacy of Contextualized Group Counseling Education in Asia: A Mixed Methods Study
Source: Int J Adv Couns. 2022 Jun 9;44(3):550–68. doi: 10.1007/s10447-022-09471-3 (PMC9178221; doi:10.1007/s10447-022-09471-3)
Supplement: Supplementary file 1 — Supplementary Material 1 [file 10447_2022_9471_MOESM1_ESM.docx]

**Highlights of the study finding**

- A contextualized experiential curriculum applied to a Malaysian group counseling education.
- Experiential learning model, integrated into Asian counseling education found effective.
- Students improved in counseling competency, leadership characteristics and critical thinking
- Students appreciated the active learning classroom, experiential activities, and feedback loop.
- The proposed training paradigm accounts for holistic thinking styles of Asian learners.
